# Social Media as Tools to Study Dietary Habits of Patients with Rheumatic Diseases: Learning from Relevant Work on Inflammatory Bowel Diseases

**DOI:** 10.31138/mjr.31.4.382

**Published:** 2020-12-22

**Authors:** Xenophon Theodoridis, Stefanos Pittas, Dimitrios P. Bogdanos, Maria G. Grammatikopoulou

**Affiliations:** 1 Department of Rheumatology and Clinical Immunology, Faculty of Medicine, School of Health Sciences, University of Thessaly, Larissa, Greece; 2 Department of Medicine, School of Health Sciences, Aristotle University of Thessaloniki, Thessaloniki, Greece; 3 Division of Transplantation Immunology and Mucosal Biology, MRC Centre for Transplantation, King’s College London Medical School, SE5 9RS, London, United Kingdom

**Keywords:** diet, social media, infodemiology, rheumatic diseases

Social media platforms have become a part of our lives, as users spend on average two and a half hours per day. Facebook, Instagram, and Twitter consist the most popular social media channels.^1^ Nowadays, more and more people are signing in to social media in order to search for medical and nutritional advices about their condition.^2^ Approximately 2/3 of Americans use social networking applications.^3^ Today, Twitter has become one of the most well-known social media platforms, with patients and health professionals exchanging opinions concerning healthcare.^4^

Due to the constantly increasing number of social media users, many scientific journals, conferences and medical societies have created accounts to propagate their research, education, events, and news, respectively.^5–7^ Currently, there are 48 twitter accounts concerning rheumatology, disseminating up-to-date recommendations and related events.^7^ However, currently, the number of tweets simultaneously containing the hashtags diet and rheumatic diseases does not exceed 20.

A recent study^8^ provides a unique opportunity to understand patients’ opinions concerning treatment options, transforming them to valuable players in decision making. The results of the study indicated that bowel disease (BD) patients turn to twitter to discuss disease symptoms, management options - including nutrition therapy -, and BD-specific comorbidities. As far as nutrition-related discussions are concerned, they tend to be focused on gluten-free diet (GFD), and dietary supplements, including probiotics and vitamin D. Furthermore, dietary interventions receive more retweets and favorites than pharmacotherapy and non-dietary interventions, making them appealing to the patient community.

Patients’ concern about their disease-related symptoms can be better reflected through social media. The severity of their symptoms, according to number of tweets, seems to differ from the objective clinical signs and symptoms that health professionals consider of high priority and examine in their daily clinical practice.^9^ The search of non-pharmacological approaches by social media users, indicate the lack of information regarding alternative treatment options than can be used as add-on therapies. Moreover, a recent study^10^ showed that direct-to-patients advertisements increase the posts, mainly regarding concerns about safety of the advertising medication, on social media platforms, reflecting the patients desire to be aware of every available treatment alternative.^11^ In patient-centered disease management, patients’ perspectives concerning their disease should be taken into consideration in the treatment algorithm, which will result in higher levels of medication adherence, satisfaction, and quality of life.^12,13^

Analyzing patients’ social media posts, provides data regarding the issues of concern on a patient’s perspective. The study of Pérez-Pérez and colleagues^8^ showed that those suffering from BD are interested in understanding the role of GFD as a possible treatment option. However, clinical practice guidelines^14^ regarding inflammatory bowel disease (IBD) management, incorporate recommendations suggesting that there is insufficient evidence to support the limitation of wheat and gluten free dietary intervention in order to ameliorate disease severity or patient symptoms. Moreover, to the best of our knowledge, no randomized controlled trials (RCTs) have been published assessing the effectiveness of adherence to a GFD in IBD. On the other hand, a recent cross-sectional study^15^ showed that patients with IBD following a GFD report improved gastrointestinal symptoms. This reveals the existence of a gap between evidence-practice and patient perceived effectiveness, indicating the need for the performance of relevant RCTs to update clinical guidelines and keep up with patient needs and interests. On the other hand, researchers can use social media platforms not only for sharing news, but also for gathering information regarding patients’ demographics characteristics, dietary habits, or for recruiting patients for either observational, or experimental studies.^1,16^ In nutrition research in particular, the use of social media has been widely exploited by researchers to record nutrition outcomes^17^ and promote healthy eating.^18,19^

Learning from the work performed by Pérez-Pérez and colleagues,^8^ researchers can exploit social media to collate information about the dietary habits of patients with rheumatic diseases, as evidence shows that dietary habits of apparently healthy controls differ from that of patients with rheumatic diseases.^20^ When understanding the dietary habits of patients with rheumatic diseases, health professionals can recommend specific lifestyle modifications by eliminating unhealthy food choices and replacing them with food items shown to reduce disease severity, such as seafood, vegetables, or fruits.^21^ For example, a recent study suggested that intermittent fasting can ameliorate psoriatic arthritis severity, defined by psoriatic arthritis disease activity scores.^22^ A review of the literature performed by Stamostergiou et al.,^23^ showed that adherence to the Mediterranean dietary pattern appears promising for the management of patients with hyperuricemia and/or gout.

In conclusion, incorporating social media in health research has many advantages and may even help in promoting evidence-based medicine.
